# Unravelling tandem repeat-mediated mutagenesis drive rapid diversification of KPC enzymes: emergence of *bla*_KPC-263_ and enhanced resistance to ceftazidime-avibactam

**DOI:** 10.1016/j.ebiom.2025.105979

**Published:** 2025-10-23

**Authors:** Haowei Ye, Ruishan Liu, Jie Shen, Wei Yang, Tongxi Hu, Xiaojing Liu, Kun Wang, Lu Gong, Hao Xu, Junfei Zhu, Zhencang Zheng, Beiwen Zheng

**Affiliations:** a State Key Laboratory for Diagnosis and Treatment of Infectious Diseases, National Clinical Research Centre for Infectious Diseases, Collaborative Innovation Centre for Diagnosis and Treatment of Infectious Diseases, The First Affiliated Hospital, Zhejiang University School of Medicine, Hangzhou, China; b Department of Critical Care Medicine, Second Affiliated Hospital, Zhejiang University School of Medicine, Hangzhou, China; c Department of Medical Oncology, The First Affiliated Hospital, College of Medicine, Zhejiang University, Hangzhou, China; d Department of Neurosurgery, The First Affiliated Hospital, College of Medicine, Zhejiang University, Hangzhou, China; e School of Basic Medical Sciences, Zhejiang Chinese Medical University, Hangzhou, China; f Jinan Microecological Biomedicine Shandong Laboratory, Jinan, China; g Department of Respiratory and Critical Care Medicine, Taizhou Central Hospital, Taizhou, China; h Yuhang Institute of Medical Science Innovation and Transformation, Hanghzou, China

**Keywords:** Tandem repeat-mediated mutagenesis, *bla*_KPC-263_, KPC variants, Ceftazidime-avibactam resistance, Carbapenem-resistant *Klebsiella pneumoniae*

## Abstract

**Background:**

The emergence of ceftazidime-avibactam (CZA)-resistant carbapenem-resistant *Klebsiella pneumoniae* (CRKP) producing *K. pneumoniae* carbapenemase (KPC) severely challenges clinical management. Understanding the genetic mechanisms underpinning their evolution, particularly the emergence of new KPC variants conferring CZA resistance, is critical.

**Methods:**

Fifteen CRKP isolates were prospectively collected in 2018 from a single patient at a tertiary-care hospital in Zhejiang, China, and were subjected to antimicrobial susceptibility testing and whole-genome sequencing. Bioinformatics analyses, including resistance gene identification, phylogenetic analysis, and comparative genomics of KPC variants and their plasmid contexts, were performed. Public NCBI databases were analysed to assess the prevalence of tandem repeat (TR)-mediated mutagenesis in KPC variants.

**Findings:**

We identified a previously uncharacterised KPC variant, *bla*_KPC-263_, resulting from a TR-mediated 3-bp insertion in the *bla*_KPC-2_ gene, which conferred enhanced resistance to CZA. A previously reported variant, *bla*_KPC-90_, with a distinct 6-bp insertion, was also isolated from the same patient. Broader genomic analysis revealed that TR-mediated mutagenesis is prevalent across 42 distinct KPC variants. Comparative plasmid analysis suggested the genetic context of *bla*_KPC-263_ may facilitate its stability.

**Interpretation:**

TR-mediated mutagenesis represents a significant, previously underappreciated genetic mechanism driving the rapid diversification and adaptive evolution of KPC enzymes under antibiotic selective pressures. These findings have broad implications for antimicrobial resistance surveillance, emphasising the need for genomic monitoring programs and targeted interventions to combat the spread of KPC variants driven by this mechanism.

**Funding:**

This work was funded by the National Key R&D Programme of China (2023YFC2308400); 10.13039/501100001809National Natural Science Foundation of China (82072314); Shandong Provincial Laboratory Project (SYS202202); Zhejiang Province Leading Geese Plan (2025C04013); Zhejiang Provincial Traditional Chinese Medicine Science and Technology Program (Grant No. 2017ZZ012); Zhejiang Provincial Medical and Health Science and Technology Project (2022KY355 & 2024KY402) and 10.13039/501100012226Fundamental Research Funds for the Central Universities (2022ZFJH003).

**Research in context**

**Evidence before this study**

Prior to this research, ceftazidime-avibactam (CZA) resistance in KPC-producing *Klebsiella pneumoniae* was a growing concern, primarily attributed to KPC variants arising from point mutations. While numerous KPC variants were known (232 in NCBI by February 2025), the role of insertional mutagenesis, particularly mediated by tandem repeats (TRs), in driving KPC evolution and CZA resistance, was largely unexplored and not well understood. The sustained isolation of multiple distinct KPC variants from a single patient was also considered rare, indicating a gap in understanding intrahost evolutionary dynamics and the full spectrum of resistance mechanisms.

**Added value of this study**

This study fundamentally expands our understanding of KPC enzyme evolution by identifying tandem repeat (TR)-mediated mutagenesis as a prevalent and clinically significant pathway for adaptive resistance to ceftazidime-avibactam. We provide a high-resolution, intra-patient account of this mechanism in action, documenting the sequential emergence of two distinct CZA-resistant variants, including the previously uncharacterised *bla*_KPC-263_, from a common ancestor under therapeutic pressure. Furthermore, our work provides a structural basis for this resistance and demonstrates through broad genomic analysis that this evolutionary strategy is not an isolated event, but is conserved across at least 42 different KPC variants, highlighting its global relevance.

**Implications of all the available evidence**

This study, combined with existing knowledge, establishes TR-mediated mutagenesis as a significant and widespread mechanism for KPC enzyme diversification and CZA resistance, impacting human health by revealing a key pathway for antibiotic evasion. These findings necessitate enhanced genomic surveillance to detect such mutations, which may be overlooked by standard methods, and underscore the importance of antibiotic stewardship. Future research should investigate the functional consequences of other TR-containing KPC variants and the precise molecular processes of TR formation. Understanding this adaptive mechanism is crucial for developing strategies to counteract resistance, address treatment failures, and improve patient outcomes in CRKP infections.

## Introduction

Carbapenem-resistant *Klebsiella pneumoniae* (CRKP) has become a global public health threat, limiting therapeutic options and increasing patient morbidity and mortality.^1^ Ceftazidime-avibactam (CZA), a β-lactam/β-lactamase inhibitor combination, has been recognised as one of the most effective antimicrobial agents for treating infections caused by *K. pneumoniae* carbapenemase (KPC)-producing strains, particularly *K. pneumoniae*, since its clinical introduction.^2^ However, the rapid emergence and dissemination of CZA-resistant KPC variants pose a significant challenge to clinical management and infection control strategies.^3^

Recent reports have documented multiple new KPC variants conferring CZA resistance, often through point mutations or amino acid substitutions within the *bla*_KPC_ gene mutations being the primary cause of resistance.^4^ The KPC variants primarily arise from amino acid substitutions, insertions, and deletions in KPC2 or KPC3,^5^ leading to alterations in protein structure and function. Previous studies have reported that KPC mutation sites are predominantly clustered in three hotspot regions: the omega loop (amino acid positions 164–179), loop 237–243, and loop 267–275.^6^ Although such mutations have been widely described, insertional mutagenesis mediated by tandem repeat (TR) represents an under-explored yet potentially critical mechanism driving resistance evolution in clinical bacterial populations. Currently, limited knowledge exists regarding the prevalence and clinical significance of TR-mediated mutagenesis in KPC enzymes, and their broader role in driving adaptive resistance evolution remains poorly understood. Moreover, while the number of reported KPC variants continues to rise, the sustained isolation of two distinct variants from the same patient remains exceptionally rare.

To address these critical gaps in our understanding of KPC evolution, this study provides a multi-level investigation into the emergence of CZA resistance. We present a rare, high-resolution analysis of intra-patient KPC diversification under direct antibiotic pressure, leading to the isolation of two distinct variants, KPC-263 and KPC-90. Crucially, we identify and characterise a previously underappreciated evolutionary pathway—tandem repeat (TR)-mediated insertional mutagenesis—as the driver for the emergence of the *bla*_KPC-263_ variant. Finally, by systematically analysing NCBI database, we assess the broader prevalence and significance of this mutational mechanism across the entire KPC enzyme family. Our findings thus reveal a widespread resistance evolution pathway with significant implications for antimicrobial resistance surveillance.

## Methods

### Clinical specimen collection, bacterial isolation and identification

Fifteen strains of CRKP were persistently isolated from clinical specimens of a 65-year-old male patient with hepatocirrhosis complicated by cholecystitis, who was hospitalised in the hepatobiliary surgery department in 2018. During the patient's hospitalisation, consecutive clinical specimens were systematically collected for microbiological examination, with specimen types including drainage fluid, sputum, bile, and blood samples.

For bacterial isolation, clinical specimens were inoculated onto blood agar and MacConkey agar plates and incubated at 37 °C for 24 h. Suspected *K. pneumoniae* colonies were selected based on morphology. Species identification for all 15 isolates was first performed using matrix-assisted laser desorption/ionization time-of-flight mass spectrometry (MALDI-TOF/MS) and subsequently confirmed by whole-genome sequencing (WGS). Clinical data, including patient demographics, infection type, and antimicrobial therapy regimens, were retrospectively obtained from electronic medical records.

### Antimicrobial susceptibility testing

Antimicrobial susceptibility testing (AST) was performed using the broth microdilution method recommended by the Clinical and Laboratory Standards Institute (CLSI) guidelines, with *Escherichia coli* ATCC 25922 as the quality control strain. The antibiotics tested included imipenem (IMP), meropenem (MEM), ceftazidime (CAZ), amikacin (AMK), ceftazidime-avibactam (CZA), colistin (CST), omadacycline, and eravacycline. AST results were interpreted according to the CLSI 2021 guidelines, while clinical breakpoints for tigecycline and colistin were based on the European Committee on Antimicrobial Susceptibility Testing (EUCAST) 2022 criteria.

### Whole-genome sequencing and bioinformatics analysis

Genomic DNA was extracted from all strains using SteadyPure Universal Genomic DNA Extraction Kit (#AG21009 Accurate Biotechnology, Hunan, China). Short-read and long-read sequences were obtained using the Illumina NovaSeq 6000 platform (Illumina, San Diego, CA, USA) and Oxford Nanopore Technologies platform (Oxford Nanopore Technologies, Oxford, UK), respectively. Prior to assembly, raw short-read data were processed using fastp v0.23.2 (https://github.com/OpenGene/fastp) to filter out low-quality reads and remove adapter contamination. Hybrid assembly of short-read and long-read sequences was performed using Unicycler v0.4.8 to generate complete genome sequences.^7^ Bacterial genomes were annotated using Prokka v1.14.5 (https://github.com/tseemann/prokka).^8^ Multilocus sequence typing (MLST) was performed using mlst (https://github.com/tseemann/mlst) for sequence type determination. Antimicrobial resistance genes were analysed using Resfinder on the CGE server (https://www.genomicepidemiology.org/services/) with a 98% identity threshold, except for KPC variants which required 100% identity for identification.^9^ Single-nucleotide polymorphism (SNP) analysis was conducted using snp-dists v0.8.2 with default parameters (https://github.com/tseemann/snp-dists). A phylogenetic tree was constructed based on core genome SNPs using Gubbins v3.4 (https://github.com/nickjcroucher/gubbins?tab=readme-ov-file), with subsequent visualisation performed in iTOL (https://itol.embl.de/).^10^ Gene copy numbers were estimated using the Carbapenemase Coding Gene Copy Number Estimator (CCNE) (https://github.com/biojiang/ccne).

### Plasmid conjugation experiments

The conjugation assay was performed according to established protocols from previous studies.^11^ Briefly, *E. coli* 600 was used as the recipient strain. Transconjugants were selected on Mueller-Hinton agar supplemented with 200 mg/L rifampicin (Rif) and 50 mg/L ampicillin (Amp). The presence of *bla*_KPC_ was confirmed by polymerase chain reaction (PCR), with additional validation using matrix-assisted laser desorption/ionization time-of-flight mass spectrometry (MALDI-TOF/MS) as previously described.^12^

### Quantitative real-time PCR (qRT-PCR) analysis of gene copy numbers

Relative expression of *bla*_KPC_ in *K. pneumoniae* strains 72478 and 73003 was evaluated by qRT-PCR, normalised to the housekeeping gene *pgi*. Total RNA was extracted from bacterial cells in logarithmic growth phase using the SteadyPure Quick RNA Extraction Kit (#AG21023 Accurate Biotechnology, Hunan, China), followed by reverse transcription into cDNA. qRT-PCR was performed using 2X SYBR Green *Pro Taq* HS Premix II (#AG11702 Accurate Biotechnology, Hunan, China) on a ViiA 7 Real-Time PCR System (Thermo Fisher Scientific, MA, USA). Primer sequences are listed in Supplementary Table S1.

### KPC carbapenemase purification and enzyme kinetic measurements

The coding sequences of *bla*_KPC-2_, *bla*_KPC-90_, and *bla*_KPC-263_ were cloned into the pET-28a vector (#B540183 Sangon Biotech, Shanghai, China) and transformed into *E. coli* BL21 (DE3) competent cells for protein expression. The KPC carbapenemases were subsequently purified to a final concentration of 1–1.5 mg/mL. Steady-state kinetic parameters (K_m_ and k_cat_) were determined by measuring the hydrolysis of β-lactam substrates, including ceftazidime (260 nm), imipenem (300 nm), and meropenem (298 nm). The data were fitted to the Michaelis–Menten equation using nonlinear regression. The half-maximal inhibitory concentration (IC_50_) values for avibactam were determined by measuring the inhibition of ceftazidime hydrolysis, and the data were analysed in GraphPad Prism 10 (GraphPad Software, MA, USA) using a dose–response inhibition, variable-slope (four-parameter) model.

### Protein structure prediction and molecular docking

Based on *bla*_KPC_ nucleotide sequences obtained from third-generation sequencing, amino acid sequences were translated and subjected to homology modelling using SWISS-MODEL (https://swissmodel.expasy.org/)with the KPC-2 crystal structure (PDB ID: 5JU3) as template,^13^ generating predicted protein structures. Energy minimisation and structural refinement of the predicted models were performed using GROMACS.^14^ Structural validation was subsequently conducted to ensure absence of backbone dihedral angle anomalies using Ramachandran plot analysis. Molecular docking simulations were performed using AutoDock Vina between the refined protein structure and each ligand (ceftazidime and avibactam) separately.^15^

### Cloning experiment

The *bla*_KPC_ gene and its associated promoter region were amplified using specific primers. The PCR products were purified and cloned into the pHSG398 vector. Recombinant plasmids pHSG398_KPC-263 and pHSG398_KPC-90 were transformed into *E. coli* DH5α competent cells via electroporation. Transformants were selected on media containing 50 mg/L chloramphenicol (Cm) and 50 mg/L ampicillin (Amp), with subsequent confirmation by PCR amplification.

### Growth curve analysis

To investigate bacterial growth under antibiotic-free conditions, growth curves were determined according to previously described methods.^16^ The tested strains included *K. pneumoniae* 70251, 72478, 73003, and the transformants DH5α_pHSG398, DH5α_pHSG398_KPC-263, and DH5α_pHSG398_KPC-90. Growth curves were plotted using R v4.3.1 based on OD_600_ measurements, with relative growth rates and area under the curve (AUC) calculated accordingly.

### Plasmid stability assay

The stability of the *bla*_KPC_-harbouring plasmid was evaluated by serially passaging the clinical isolate in antibiotic-free Luria–Bertani (LB) broth for 15 consecutive days. The culture was diluted 1:1000 daily, corresponding to approximately 150 generations of growth without selective pressure. At 3-day intervals, 30–50 colonies were randomly selected from plated cultures and screened by PCR for the presence of the *bla*_KPC_ and *rep* genes. The entire experiment was performed in triplicate.

### Statistical analysis

To compare quantitative data from the bacterial fitness experiments, such as the area under the growth curve (AUC), a one-way analysis of variance (ANOVA) was performed. This method was chosen to assess differences in mean growth across three or more independent experimental groups. Following the ANOVA, Šídák's multiple comparisons test was used as a post-hoc analysis to conduct specific pairwise comparisons between groups and identify which differences were statistically significant. All experiments for the growth analysis were conducted in three replicates. Significance levels will be indicated by ∗P < 0.05, ∗∗P < 0.01, ∗∗∗P < 0.001 and ∗∗∗∗P < 0.0001.

### Role of funders

The funders had no role in the study's design, data collection, data analyses, interpretation, or writing of manuscript.

## Results

### Longitudinal intrahost pathogen tracking during therapy

**Fig. 1:**
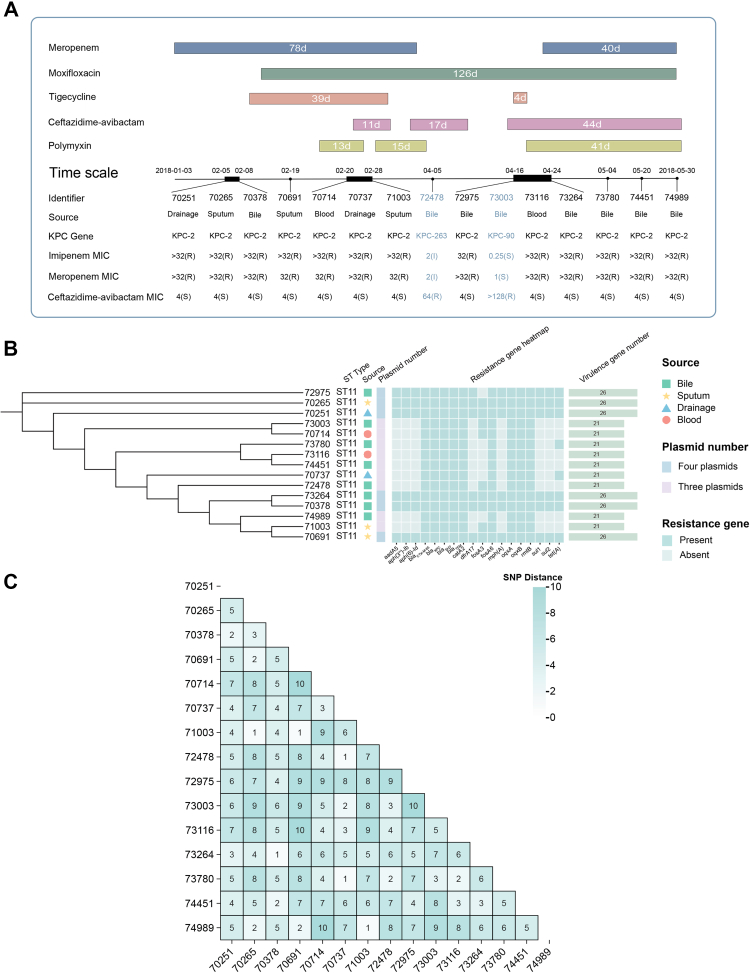
(A) Timeline of clinical antimicrobial treatment regimens administered to the patient and corresponding collection dates for the 15 *K. pneumoniae* isolates analysed in this study. Antibiotic treatment periods and key therapeutic changes (including initiation, discontinuation, and combination therapy adjustments) are highlighted, clearly illustrating correlations between antibiotic regimens and the emergence of resistance phenotypes. (B) Phylogenetic tree and related information of all strains. (C) Heatmap visualisation of single-nucleotide polymorphism (SNP) variations among 15 *K. pneumoniae* clinical isolates. Numerical values indicate pairwise SNP distances between strains, with colour gradients representing magnitude differences. The heatmap highlights closely related strain clusters, suggesting strain evolution under selective antibiotic pressures within the patient.

All *K. pneumoniae* isolates were obtained from a single patient admitted to a tertiary-care hospital in Zhejiang, China. Over the course of hospitalisation, a total of 15 consecutive clinical specimens, including drainage fluid, sputum, bile, and blood samples, were systematically collected for microbiological examination. Initially, isolates 70251, 70265, and 70378 displayed resistance to carbapenems (MEM, IPM; MICs ≥32 mg/L), which correlated with meropenem monotherapy (1 g q8h). After 17 days of CZA monotherapy (2.5 g q8h), the initial CZA-resistant *K. pneumoniae* strain 72478 was recovered from bile specimens. One week after CZA withdrawal, a CZA-susceptible *K. pneumoniae* strain 72975 was re-isolated from bile. However, upon resumption of CZA therapy, a second CZA-resistant strain 73003 re-emerged from bile specimens. Subsequent combination therapy with imipenem and CZA resulted in isolation of *K. pneumoniae* strains (73264, 73780, 74451, 74989) exhibiting meropenem resistance but restored susceptibility to CZA. A detailed timeline of antimicrobial therapy and corresponding strain isolation events is presented in Fig. 1A.

### Dynamic resistance modulation in *K. pneumoniae* under antibiotic selection pressure

Antimicrobial susceptibility testing revealed that all clinical *K. pneumoniae* isolates exhibited high-level resistance to aztreonam, ceftazidime, amikacin and other tested antimicrobials (Table 1). Specifically, *K. pneumoniae* strains 72478 and 73003 demonstrated high resistance to CZA with MICs of 64 mg/L and >128 mg/L, respectively, while remaining susceptible to meropenem (MEM) and imipenem (IPM). Conversely, all other strains showed high resistance to MEM and IPM (MICs ≥32 mg/L), while remaining susceptible to CZA. Importantly, we observed dynamic shifts in resistance profiles directly linked to the patient's antimicrobial regimen adjustments. Notably, CZA monotherapy rapidly led to the selection and emergence of CZA-resistant isolates, whereas temporary CZA discontinuation resulted in transient restoration of susceptibility. Subsequent CZA reintroduction rapidly selected resistant strains, confirming the highly adaptive nature of *K. pneumoniae* under selective antibiotic pressure. These findings underscore the critical need for cautious use and continuous monitoring of new antimicrobials such as CZA to prevent the rapid emergence and dissemination of resistant variants.

**Table 1:** MICs of all original strains and cloned strains.

Isolates	MIC (mg/L)									
	IPM	MEM	CAZ	AMK	ATM	CZA	TGC	CST	Omadacycline	Eravacycline
70251 (KPC-2)	32 (R)	32 (R)	>128 (R)	>128 (R)	>128 (R)	4 (S)	8 (R)	0.5 (S)	>32 (R)	8 (R)
70265 (KPC-2)	>32 (R)	>32 (R)	>128 (R)	>128 (R)	>128 (R)	4 (S)	8 (R)	0.25 (S)	>32 (R)	8 (R)
70378 (KPC-2)	>32 (R)	>32 (R)	>128 (R)	>128 (R)	>128 (R)	4 (S)	8 (R)	0.25 (S)	>32 (R)	8 (R)
70691 (KPC-2)	32 (R)	32 (R)	>128 (R)	>128 (R)	>128 (R)	4 (S)	8 (R)	0.5 (S)	>32 (R)	8 (R)
70714 (KPC-2)	32 (R)	32 (R)	>128 (R)	>128 (R)	>128 (R)	4 (S)	2 (S)	0.5 (S)	32 (R)	2 (R)
70737 (KPC-2)	32 (R)	32 (R)	>128 (R)	>128 (R)	>128 (R)	4 (S)	1 (S)	>32 (R)	8 (I)	1 (R)
71003 (KPC-2)	32 (R)	32 (R)	>128 (R)	>128 (R)	>128 (R)	4 (S)	2 (S)	0.25 (S)	>32 (R)	4 (R)
72478 (KPC-263)	2 (I)	2 (I)	>128 (R)	>128 (R)	>128 (R)	64 (R)	0.5 (S)	0.25 (S)	8 (I)	0.5 (S)
72975 (KPC-2)	32 (R)	>32 (R)	>128 (R)	>128 (R)	>128 (R)	4 (S)	8 (R)	0.25 (S)	>32 (R)	8 (R)
73003 (KPC-90)	0.25 (S)	1 (S)	>128 (R)	>128 (R)	>128 (R)	>128 (R)	0.5 (S)	0.5 (S)	4 (S)	0.5 (S)
73116 (KPC-2)	32 (R)	32 (R)	>128 (R)	>128 (R)	>128 (R)	4 (S)	4 (I)	0.5 (S)	>32 (R)	8 (R)
73264 (KPC-2)	>32 (R)	>32 (R)	>128 (R)	>128 (R)	>128 (R)	4 (S)	4 (I)	>32 (R)	>32 (R)	8 (R)
73780 (KPC-2)	>32 (R)	>32 (R)	>128 (R)	>128 (R)	>128 (R)	4 (S)	2 (S)	0.5 (S)	>32 (R)	4 (R)
74451 (KPC-2)	>32 (R)	>32 (R)	>128 (R)	>128 (R)	>128 (R)	4 (S)	2 (S)	16 (R)	>32 (R)	4 (R)
74989 (KPC-2)	>32 (R)	>32 (R)	>128 (R)	>128 (R)	>128 (R)	4 (S)	2 (S)	16 (R)	>32 (R)	4 (R)
*E. coli* DH5α	0.125 (S)	≤0.25 (S)	0.5 (S)	≤1 (S)	0.125 (S)	0.125 (S)	0.125 (S)	0.25 (S)	2 (S)	0.125 (S)
*E. coli* DH5α_pHSG398	0.125 (S)	≤0.25 (S)	0.5 (S)	≤1 (S)	0.125 (S)	0.125 (S)	≤0.06 (S)	0.25 (S)	2 (S)	0.125 (S)
*E. coli* DH5α_pHSG398_KPC-263	0.125 (S)	≤0.25 (S)	>32 (R)	≤1 (S)	4 (S)	8 (S)	≤0.06 (S)	0.25 (S)	2 (S)	0.125 (S)
*E. coli* DH5α_pHSG398_KPC-90	0.125 (S)	≤0.25 (S)	>32 (R)	≤1 (S)	4 (S)	16 (R)	≤0.06 (S)	0.25 (S)	2 (S)	0.125 (S)

IPM, imipenem; MEM, meropenem; CAZ, ceftazidime; AMK, amikacin; ATM, aztreonam; CZA, ceftazidime-avibactam; TGC, tigecycline; CST, colistin. For ceftazidime/avibactam, the avibactam was tested at a fixed concentration of 4 mg/L.

### Genomic relatedness and SNP-based evolutionary analysis

Whole-genome sequencing of all 15 isolates identified them as ST11-KL47 *K. pneumoniae* (Fig. 1B), a prevalent and clinically significant clone that is widely disseminated and frequently reported as a cause of hospital-acquired infections. To determine phylogenetic relationships, we performed pairwise SNP analysis of core genomes. Core genome comparison revealed 1–10 SNPs between chromosomal sequences of all isolates, demonstrating high genetic relatedness (Fig. 1C). Heatmap visualisation clearly demonstrated a close genomic relatedness among isolates (pairwise SNP distances ≤20), indicative of in-patient evolutionary events rather than multiple external introductions. Notably, isolates resistant to CZA clustered closely, suggesting recent evolutionary events driven by antibiotic selective pressures.

### Identification and genomic characterisation of *bla*_KPC-263_ and *bla*_KPC-90_

Analysis of resistance gene profiles from assembled genomes revealed the presence of multiple resistance determinants. All isolates contained multiple resistance determinants: β-lactamase genes (*bla*_SHV-12_, *bla*_TEM-1B_, *bla*_CTX-M-65_), aminoglycoside resistance genes (*aadA5*, *rmtB*), fosfomycin resistance gene *fosA6*, fluoroquinolone resistance gene *oqxB*, macrolide resistance gene *mph(A)*, and tetracycline resistance gene *tet(A)*. Crucially, a previously uncharacterised KPC variant (designated KPC-263) was identified in isolate 72478, resulting from a unique 3-bp TR insertion in *bla*_KPC-2_. Moreover, a KPC variant, KPC-90, was observed in isolate 73003, which was mediated by a 2-amino-acid insertion outside the KPC omega-loop region. This highlights the co-occurrence and possible intrahost evolution of KPC variants arising through different mutational mechanisms, including TR-mediated insertion (KPC-263) and unrelated insertions (KPC-90).

### Sequence characterisation of *bla*_KPC-263_ and *bla*_KPC-90_

**Fig. 2:**
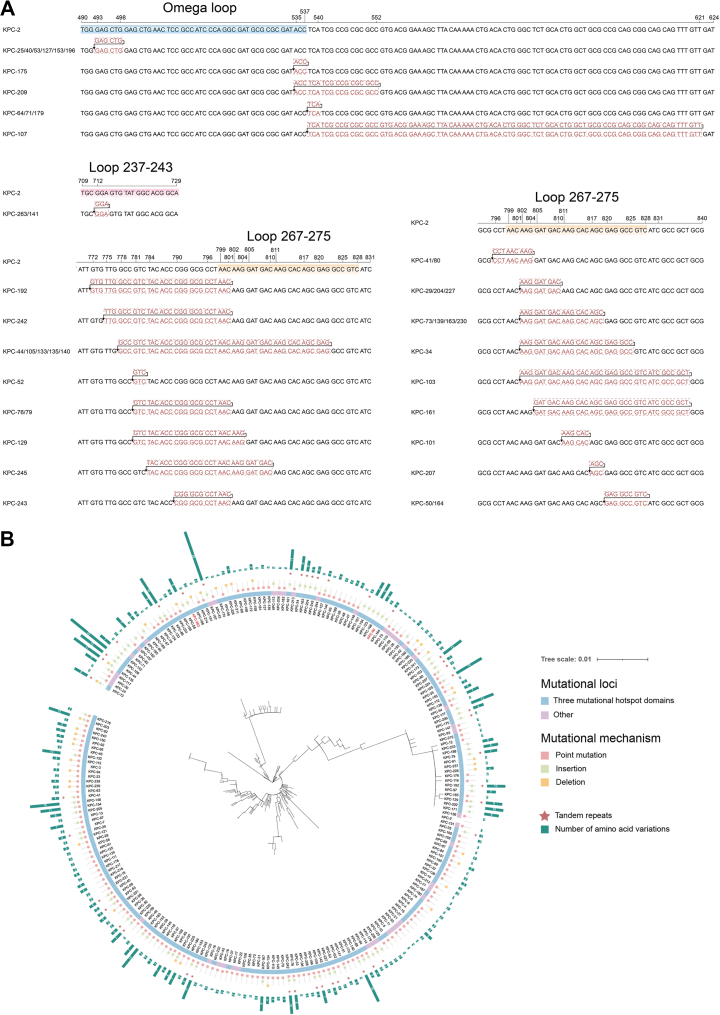
(A) Sequence alignment of 42 KPC variants from NCBI compared with KPC-2. Red font indicates inserted sequences and TR formed. Sequences marked by blue, pink, and yellow blocks represent three mutation hotspot regions: omega loop, loop 237–243, and loop 267–275. (B) Maximum-likelihood phylogenetic tree of KPC variants. Among the analysed KPC variants, 190 exhibited mutations within three defined hotspot regions, whereas 42 variants contained mutations outside these conserved areas. (The three mutational hotspots comprise the omega loop (residues 164–179), loop 237–243, and loop 267–275.) Different mutation sites and KPC variants with TRs are marked with different colour blocks and shapes, while a bar chart shows the number of amino acid variations.

Compared to *bla*_KPC-2_, *bla*_KPC-263_ exhibited a 3-bp insertion (positions 712–714) resulting in glycine insertion at codon 238 (^238^G). *bla*_KPC-90_ contained a 6-bp insertion (positions 535–540) introducing threonine–tyrosine dipeptide at codons 179–180 (^179^T^180^Y) (Fig. 2A). The *bla*_KPC-263_ insertion (GGA) constituted a tandem duplication that maintained the reading frame without causing frameshift mutation. To investigate the prevalence of tandem duplications, all KPC variant sequences (as of February 2025) were retrieved from NCBI Pathogen Detection Reference Gene Catalog (https://www.ncbi.nlm.nih.gov/pathogens/refgene/#gene_family:(blaKPC)). A phylogenetic tree was reconstructed based on single-nucleotide polymorphisms (SNPs) (Fig. 2B). To further assess the broader significance of TR-mediated mutagenesis, sequence alignment identified 42 variants with similar tandem duplications, including *bla*_KPC-25_, *bla*_KPC-71_, *bla*_KPC-164_, and *bla*_KPC-175_. Insertion lengths ranged from 3 to 84 bp (all multiples of 3), producing in-frame mutations without aberrant translational consequences. These duplications likely originated from DNA polymerase slippage during replication or defective mismatch repair mechanisms.^17^ This finding underscores that TR-driven insertional mutagenesis represents a conserved and broadly applicable evolutionary mechanism driving functional diversification among clinically relevant KPC carbapenemases.

### Kinetic characterisation reveals functional trade-offs in KPC variants

Enzymatic kinetic analysis revealed that the KPC mutations induced complex and divergent effects on substrate hydrolysis and inhibitor susceptibility (Table 2). Against ceftazidime, the two variants behaved differently: the catalytic efficiency (k_cat_/K_m_) of KPC-263 was reduced by approximately 36% compared to wild-type KPC-2, whereas no significant change was observed for KPC-90. In a striking functional trade-off, both variants exhibited a near-complete loss of hydrolytic activity against carbapenems (imipenem and meropenem), which provides an enzymatic basis for the restored carbapenem susceptibility observed in the clinical isolates. Furthermore, the mutations conferred distinct levels of reduced susceptibility to avibactam. KPC-263 showed a moderate effect, with its IC_50_ value increasing by approximately 25% relative to KPC-2. The impact was substantially more pronounced for KPC-90, which demonstrated significantly diminished susceptibility, with its IC_50_ value increasing by 117% relative to KPC-2. These results indicate that both variants have a lower affinity for avibactam than KPC-2, but this effect is markedly greater for KPC-90.

**Table 2:** Kinetic parameters and IC_50_ of purified KPC-2 and KPC variants.

Drugs	KPC-2				KPC-263				KPC-90			
	k_cat_ (s^−1^)	K_m_ (μM)	k_cat_/K_m_ (μM^−1^ s^−1^)	IC_50_ (μM)	k_cat_ (s^−1^)	K_m_ (μM)	k_cat_/K_m_ (μM^−1^ s^−1^)	IC_50_ (μM)	k_cat_ (s^−1^)	K_m_ (μM)	k_cat_/K_m_ (μM^−1^ s^−1^)	IC_50_ (μM)
Ceftazidime	42.9	580	0.074	∖	32.15	680	0.0473	∖	48.75	620	0.0786	∖
Imipenem	198.75	380	0.523	∖	ND	ND	ND	∖	ND	ND	ND	∖
Meropenem	112.36	420	0.268	∖	ND	ND	ND	∖	ND	ND	ND	∖
Avibactam	∖	∖	∖	7.0	∖	∖	∖	8.72	∖	∖	∖	15.2

ND, not determined due to subthreshold initial hydrolysis kinetics.

### Genomic characterisation of *bla*_KPC_ harbouring plasmids

**Fig. 3:**
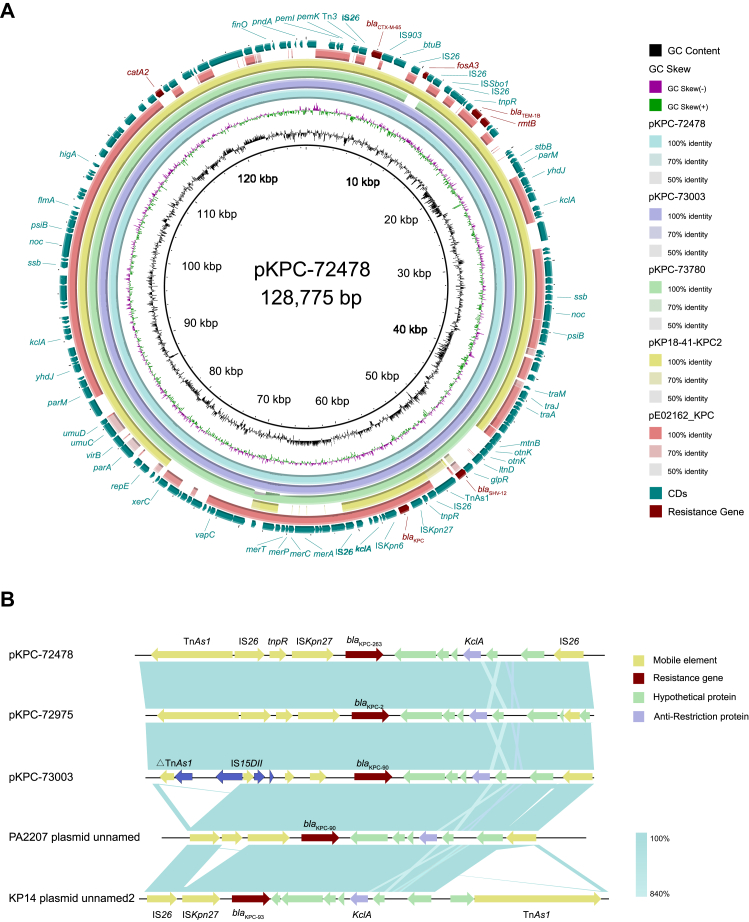
(A) Comparative genomic analysis of pKPC-72478 with closely related homologous plasmids, with pKPC-72478 serving as the reference sequence. (B) Detailed comparison of conserved genomic architecture flanking the *bla*_KPC_ gene among plasmids pKPC-72478, pKPC-72975, pKPC-73003, and previously reported plasmids PA2207 and KP14. Cyan-shaded regions represent sequence homology (84–100% identity) as determined by BLASTn comparative genomics. Predicted ORFs are denoted by arrow annotations, highlighting gene orientation and conserved structural arrangements that potentially facilitate stable maintenance and dissemination of *bla*_KPC_ variants.

Detailed comparative genomic analysis was performed on the plasmids harbouring *bla*_KPC-263_ and related variants. Plasmid sequences were obtained for all isolates (Supplementary Table S2). All isolates carried multiple plasmid types including IncFIB, IncR, IncFII, and ColRNAI. Based on SNP and phylogenetic analysis of all strains, these isolates showed high homology to each other. The plasmid pKPC-72478 was characterised as an IncR/FII-type plasmid (∼128 kb), closely related to plasmids identified in other clinically significant Enterobacterales. Comparative genomics revealed a highly conserved genetic backbone shared by *bla*_KPC_-harbouring plasmids from this study and previously sequenced plasmids such as pKP18-41-KPC2 (GenBank: CP082012) and pE02162_KPC (GenBank: MK370991), indicating strong evolutionary pressures to retain critical resistance determinants within stable plasmid contexts (Fig. 3A). Plasmids pKP18-41-KPC2 and pE02162_KPC shared similar IS*26*-*tnpR*-IS*Kpn27*-*bla*_KPC_-IS*Kpn6*-*KclA*-IS*26* configurations, though not identical to pKPC variants in this study. Notably, IS*26*-mediated deletion of *fosA3* was observed in pKPC-73780. Comparative analysis revealed minor structural variations caused by partial sequence loss of mobile elements including IS*26*.

As shown in Fig. 3B, the genetic context of *bla*_KPC-263_ was characterised as Tn*As1*-IS*26*-*tnpR*-IS*Kpn27*-*bla*_KPC_-IS*Kpn6*-*KclA*-IS*26*, while *bla*_KPC-90_ resided within a △Tn*As1*-IS*15DII*-*tnpR*-IS*Kpn27*-*bla*_KPC_-IS*Kpn6*-*KclA*-IS*26* structure. Further analysis demonstrated genetic context diversity: *bla*_KPC-93_ in KP14 plasmid unnamed2 (GenBank: CP087153) was flanked by IS*26*-IS*Kpn27*-*bla*_KPC-93_-*KclA-*Tn*As1*, whereas *bla*_KPC-90_ in PA2207-plasmid-unnamed (GenBank: CP080290) exhibited IS*26*-*tnpR*-IS*Kpn27*-*bla*_KPC-90_-IS*Kpn6*-*KclA*-IS*26* organisation. These configurations differed from the canonical Tn*4401* transposon structure.^18^ Crucially, transposable elements were found to play a pivotal role in the genetic diversification and evolution of *bla*_KPC_ variants.^19^

No transconjugants were obtained in three replicate experiments conducted under standard (37 °C) or low-temperature (25 °C) conditions. The oriTDB result showed there no origin site of DNA transfer (*oriT*), relaxase, type IV coupling protein (T4CP) and type IV secretion system (T4SS) were found in the pKPC-72478 and pKPC-73003.^20^ The genomic architecture of both pKPC-72478 and pKPC-73003 lacked essential conjugative transfer elements, providing the molecular basis for the negative results obtained in conjugation experiments.

### Molecular docking and structural features of KPC variants

The structural evolution of carbapenemases under antimicrobial selection pressure represents a critical frontier in understanding resistance adaptation, where ligand-binding landscape remodelling may recalibrate enzyme-inhibitor affinities to subvert therapeutic efficacy. To dissect how allelic diversification among KPC variants structurally reprogrammes β-lactamase inhibition, we conducted comparative molecular docking simulations across KPC-2, KPC-90, and KPC-263, resolving variant-specific ligand interaction architectures that mechanistically decode resistance trajectories. Binding affinity results are presented in Supplementary Table S3, indicating the relative binding strength between ligands and enzyme variants.^21^ Both variants exhibited reduced binding affinities for CAZ and AVI compared to KPC-2, with more pronounced reduction observed for AVI, suggesting enhanced resistance to the β-lactamase inhibitor. This correlates with the elevated resistance to CZA observed in strains harbouring these variants.

**Fig. 4:**
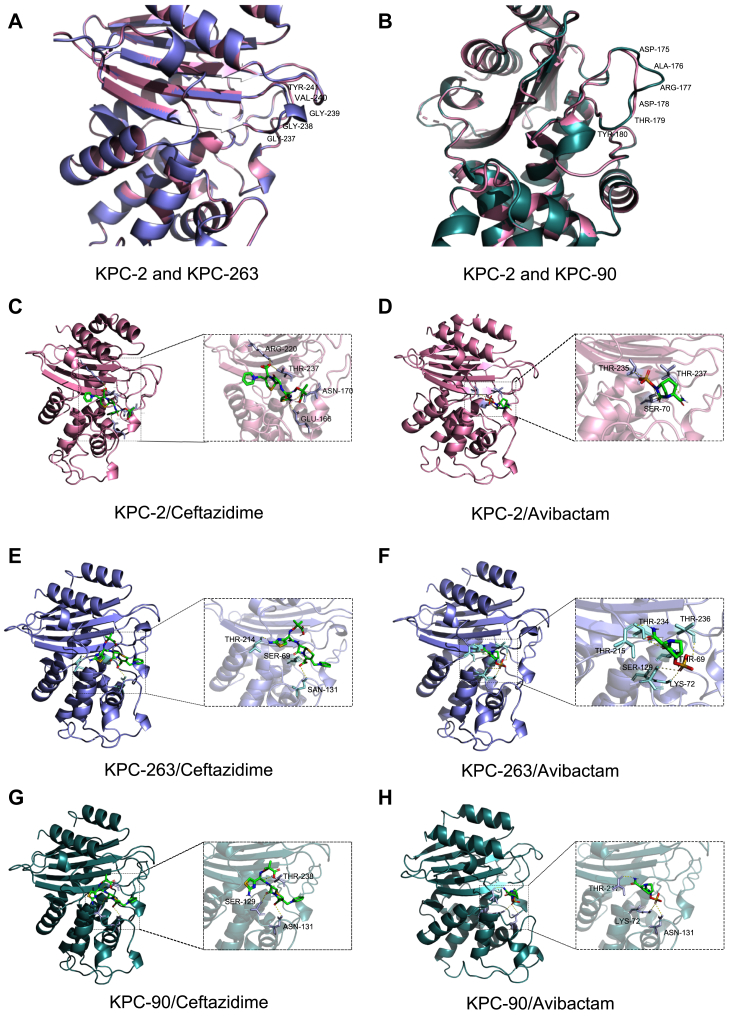
(A) Protein structure comparison between KPC-2 and KPC-263. (B) Protein structure comparison between KPC-2 and KPC-90. (C-D) Molecular docking diagram of KPC-2 with ceftazidime/avibactam. (E-F) Molecular docking diagram of KPC-263 with ceftazidime/avibactam. (G-H) Molecular docking diagram of KPC-90 with ceftazidime/avibactam.

As shown in Fig. 4A–H, the spatial arrangement of ceftazidime's isothiazole and pyridine rings critically influenced binding interactions with KPC enzymes. The glycine insertion at position 238 in KPC-263 induced conformational changes in the backbone structure compared to KPC-2. Structural analysis revealed that the ^238^G insertion promoted α-helix formation in the 237–243 loop region, resulting in upward displacement of residues 235–237 and downward shift of 240–242 residues, with concomitant contraction of the 236–242 loop (Fig. 4A). In KPC-90, the ^179^T^180^Y dipeptide insertion caused expansion of the random coil conformation within residues 169–182 (Fig. 4B). These structural modifications altered key catalytic residues, thereby affecting ligand binding affinities (Fig. 4C–H). Collectively, the structural adaptations in KPC-263 and KPC-90 enhance ceftazidime hydrolysis while reducing avibactam efficacy, ultimately conferring resistance to CZA.

### Fitness cost and plasmid stability of KPC variants

**Fig. 5:**
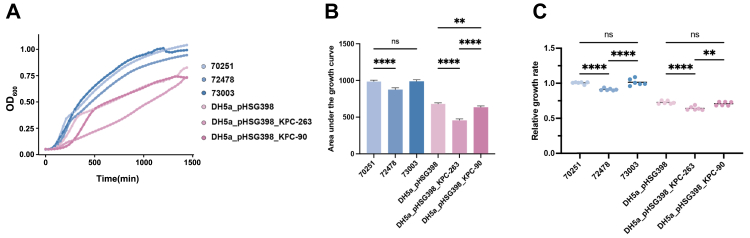
(A) Growth curves of clinical isolates and recombinant strains carrying *bla*_KPC_, (B) area under growth curve, (C) relative growth rate. Error bars represent standard deviation from three replicates. Statistical significance was determined by a one-way ANOVA followed by Šídák's multiple comparisons test. ∗P < 0.05; ∗∗P < 0.01; ∗∗∗∗P < 0.0001 (Šídák's multiple comparisons test).

To investigate growth effects conferred by *bla*_KPC_ variants, bacterial fitness was assessed under antibiotic-free conditions through growth curve analysis, area under the curve (AUC), and relative growth rate calculations. *K. pneumoniae* 72478 exhibited significantly lower growth rates compared to strains 70251 and 73003, while no significant difference was observed between 70251 and 73003. Furthermore, comparative analysis of transformants revealed that *E. coli* DH5α_pHSG398_KPC-263 showed reduced growth rates and decreased AUC values relative to *E. coli* DH5α_pHSG398_KPC-90 and the empty vector control (Fig. 5A–C). These findings suggest that *bla*_KPC-90_ imposes lower fitness costs on bacterial hosts compared to *bla*_KPC-263_, potentially accounting for the selection of *bla*_KPC-90_-carrying *K. pneumoniae* 73003 (CZA-resistant) over *bla*_KPC-263_-harbouring variants during later isolation phases.

In addition to growth kinetics, we performed a 15-day serial passage experiment to assess the stability of the native IncFII/IncR plasmids carrying the *bla*_KPC_ variants. The plasmids in both *K. pneumoniae* 72478 and *K. pneumoniae* 73003 remained highly stable, with retention rates exceeding 90% after approximately 150 generations of growth without antibiotic selection (Suppelmentary Figure S1). This indicates that the presence of the KPC variant insertions did not significantly compromise plasmid stability in the host.

## Discussion

The emergence and global dissemination of CRKP have significantly challenged clinical treatment regimens, prompting the development and clinical adoption of new antimicrobial agents such as CZA.^22^ However, the rapid appearance of resistance to CZA among KPC-producing strains has complicated infection management, underscoring the urgent need for understanding underlying resistance mechanisms. Here, we report a previously underrecognized resistance mechanism—TR-mediated insertional mutagenesis—in *bla*_KPC_ genes, driving the rapid evolution of resistance to CZA in clinical isolates of *K. pneumoniae*.

Our study revealed the emergence of *bla*_KPC-263_, a previously uncharacterised KPC variant characterised by a unique 3-bp TR insertion, derived from the ancestral *bla*_KPC_ allele. In contrast, KPC-90, found in isolate 73003, harboured a 2-amino-acid insertion located outside the canonical omega-loop region, but was not associated with TR. Cloning experiment confirmed that the presence of this insertion directly contributes to a moderate increase in resistance to CZA (MIC = 8 mg/L). It is noteworthy that while the KPC-263 enzyme alone conferred this level of non-susceptibility in an *E. coli* cloning host, the original clinical isolate 72478 exhibited a much higher level of resistance (MIC = 64 mg/L). This eight-fold difference in MIC can be attributed to synergistic resistance mechanisms present in the *K. pneumoniae* host. Specifically, our genomic analysis of isolate 72478 revealed the complete deletion of the OmpK35 porin gene and ^134^G^135^D dipeptide insertion in the L3 loop of the OmpK36 porin (Supplementary Figure S2). The loss of OmpK35 and alteration of OmpK36 are known to significantly reduce the influx of β-lactam antibiotics, including ceftazidime.^23^ Therefore, the high-level resistance observed in the clinical isolate is unequivocally multifactorial, resulting from the combined effect of reduced drug permeability due to porin modifications and enhanced hydrolysis by the new KPC-263 variant. Interestingly, KPC-90 was also recovered from the same patient who harboured KPC-263. Compared with KPC-263, KPC-90 contained a longer insertion within the *bla*_KPC-2_ gene and demonstrated higher resistance to ceftazidime-avibactam (MIC = 16 mg/L). This phenotypic discrepancy underscores the critical role of TR length and location in modulating KPC enzymatic activity. Meanwhile, quantitative PCR and carbapenemase gene copy number estimation revealed no *bla*_KPC_ copy number amplification associated with this resistance phenotype (Supplementary Figure S3). This intrahost co-occurrence of functionally convergent yet mechanistically distinct KPC variants provides compelling evidence of adaptive diversification under selective antibiotic pressure. Notably, our comparative genomic analysis and structural context suggest that *bla*_KPC_ evolution is not restricted to a single mutational pathway. Instead, it may proceed via multiple distinct genetic events—including TR-mediated mutagenesis and independent small indels—resulting in altered β-lactamase activity and differential drug resistance phenotypes. Such findings not only underscore the remarkable plasticity of carbapenemase genes within a single host but also highlight the need for comprehensive surveillance strategies that account for diverse and parallel resistance evolution pathways.

To assess the broader relevance of our findings beyond a single clinical case, we conducted a comprehensive analysis of publicly available *bla*_KPC_ sequences in the NCBI database. Remarkably, we identified at least 42 distinct KPC variants (including KPC-25, KPC-71, KPC-107) containing similar in-frame TR insertions (Fig. 2A). These insertions, ranging from 3 to 84 bp, maintained tri-nucleotide periodicity, preserving the reading frame without major structural disruption to KPC proteins. This strongly suggests that TR-mediated mutagenesis is not a sporadic event, but rather a widespread and conserved mechanism contributing to the functional diversification and adaptive evolution of KPC enzymes under antimicrobial selective pressure.

**Fig. 6:**
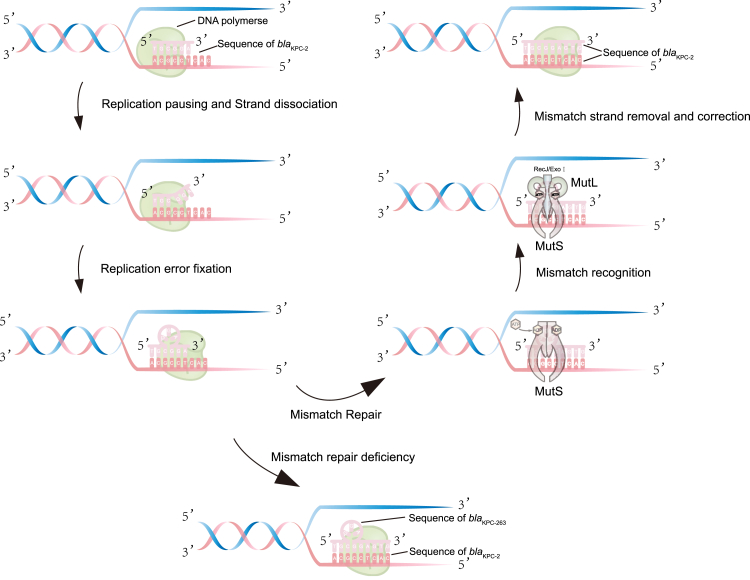
Proposed model for the emergence of the *bla*_KPC-263_ variant via tandem repeat-mediated mutagenesis under mismatch repair (MMR) control. Post DNA slippage replication, schematic representations of two possible outcomes: functional MMR and MMR deficiency. The MutS protein recognises mismatched bases or insertion/deletion loops (IDLs) in DNA. MutL interacts with MutS to facilitate recruitment of downstream repair factors. The erroneous strand is excised by RecJ/ExoⅠ nucleases, followed by resynthesis of the correct sequence by DNA polymerase. In contrast, under MMR-deficient or stress-compromised conditions, erroneous repeat insertions may escape repair, leading to stable in-frame TR expansions such as the 3-bp insertion observed in *bla*_KPC-263_. This model is speculative and intended to illustrate a plausible mechanism underlying the intrahost emergence of KPC variants.

Mechanistically, short TR (≤6 bp), such as the 3-bp repeat in KPC-263, likely arose through slipped-strand mispairing (SSM) during DNA replication,^24^^,^^25^ involving bulge formation on nascent strands. As shown in Fig. 6, during DNA replication, the template strand and the newly synthesised strand undergo local unwinding and mismatching, leading to a bulge in the nascent strand and resulting in short TR insertion.^25^ When the mismatch repair (MMR) system is functional, the MutS protein recognises mismatched bases or insertion/deletion loops (IDLs) in the DNA. MutL cooperates with MutS to recruit downstream repair proteins. Guided by the MutS–MutL complex, MutH cleaves the unmethylated new strand, RecJ/ExoⅠ degrades the incorrect strand, and DNA polymerase resynthesises the correct sequence.^26^^,^^27^ However, when the MMR system is defective, the insertion mutation cannot be recognised and repaired, leading to the generation of mutants.

In contrast, larger insertions (>10 bp), as observed in KPC-107 and related variants, likely arise through microhomology-mediated break-induced replication (MMBIR),^28^ where replication fork collapse at GC-rich regions triggers DNA polymerase θ (Polθ)-mediated repair—a process involving microhomology alignment (2–5 bp) at stalled forks, template switching, and error-prone strand synthesis.^29^^,^^30^ The intrinsic low fidelity of Polθ exacerbates TR generation during gap-filling, particularly when flanked by mobile elements, while secondary structure formation further stabilises insertion intermediates. This mechanism contrasts with short TR (≤6 bp) generated via slipped-strand mispairing.

Collectively, these findings provide systematic evidence that TR-mediated mutagenesis constitutes a generalisable evolutionary strategy exploited by *K. pneumoniae* to expand the functional repertoire of KPC enzymes. These discoveries not only broaden our understanding of resistance development at the population level but also highlight the urgent need for genomic surveillance systems capable of detecting these cryptic, structurally subtle yet functionally significant mutations.

While TR in antimicrobial resistance genes remain understudied, emerging evidence suggests their critical role in bacterial adaptation.^17^^,^^24^^,^^31^ In *Pseudomonas aeruginosa*, TR variations upstream of *oprD* disrupt promoter regions or translation initiation sites, leading to *oprD* silencing and carbapenem resistance.^32^ *Candida albicans* demonstrates *ERG11* promoter microsatellite variations that upregulate gene expression and confer fluconazole resistance.^33^ Distinctively, the in-frame TR identified in *bla*_KPC_ variants (with tri-nucleotide periodicity) induces domain-specific conformational changes that optimise antibiotic resistance phenotypes under selective pressures. These paradigmatic cases across bacterial and fungal pathogens illuminate TRs as molecular rheostats of genomic plasticity, where localised sequence reiterations fine-tune resistance determinants through divergent yet precise mechanisms—from transcriptional silencing (*oprD*) to promoter hyperactivation (ERG11) and allosteric domain remodelling (*bla*_KPC_). Such TR-driven adaptive mutations exemplify pathogen evolutionary economisation: minimal genetic alterations maximising phenotypic payoffs under antimicrobial stress. Decrypting these repeat-mediated resistance blueprints demands convergent structural biology and pan-genomic surveillance to counteract microbial escape routes in the post-genomic era.

All *bla*_KPC_ genes in this study resided on IncFII/IncR hybrid plasmids, which was reported to be one of the key vectors mediating transmission of *bla*_KPC_,^34^ demonstrating enhanced structural stability but reduced transmissibility compared to globally circulating counterparts.^35^ IS*26*-mediated recombination events were consistently associated with structural variations in pKPC plasmids, suggesting this mobile element drives both resistance diversification and dissemination heterogeneity.^36^ Distinct from the canonical Tn4401 transposon, evolutionary analysis revealed multiple core configurations flanking *bla*_KPC_ variants: IS*Kpn6*-*bla*_KPC_-IS*Kpn28*, *tnpR*-*tnpA*-IS*Kpn7*-*bla*_KPC_-IS*Kpn6*, and IS*Kpn27*-*bla*_KPC_-IS*Kpn6*.^37^ The previously uncharacterised *bla*_KPC-263_ and *bla*_KPC-90_ variants exhibited a unique *tnpR*-IS*Kpn27*-*bla*_KPC_-IS*Kpn6* core structure bracketed by IS*26* elements at both termini. Despite lacking conventional conjugation machinery, IS*26* facilitated KPC dissemination through replicative cointegrate formation between donor and recipient molecules, enabling horizontal gene transfer across bacterial species.^38^^,^^39^ These findings highlight the pivotal role of IS*26* in shaping *bla*_KPC_ plasmid evolution, driving both structural diversification and dissemination despite limited transmissibility.

This investigation yields significant insights into TR-mediated mutagenesis as a crucial mechanism driving KPC enzyme diversification and resistance to ceftazidime-avibactam, though certain constraints merit consideration. Firstly, the clinical evaluation was confined to an individual patient case, potentially restricting the broader applicability of the documented intrahost evolutionary patterns. Secondly, while conserved genetic environments theoretically conducive to dissemination were identified, conjugation assays unexpectedly failed to demonstrate plasmid transfer, implying either methodological constraints in our experimental approach or as-yet-unidentified obstacles to horizontal gene transfer. Thirdly, our structural conclusions rely on computational modelling; while validated by Ramachandran plot analysis, these predictions require definitive confirmation of conformational changes through future crystallographic studies.

Further limitations also warrant discussion. Although our study characterises the enzymatic and structural properties of the KPC-263 variant, its precise impact on pathogenesis and clinical outcomes requires validation in future *in vivo* animal infection models. Additionally, the global effect of the KPC mutations on bacterial physiology remains to be fully elucidated, as comparative transcriptomic and proteomic analyses were not performed. Finally, while KPC-90 was functionally characterised, the specific evolutionary dynamics and selective pressures that favoured its emergence over the KPC-263 variant within the host present a complex area for further investigation.

In summary, our work elucidates TR-mediated mutagenesis as a key evolutionary mechanism driving the rapid diversification of KPC carbapenemases, exemplified by the previously uncharacterised *bla*_KPC-263_ variant and its association with CZA resistance. Our comprehensive genomic analysis reveals this mechanism's widespread prevalence across 42 distinct KPC variants, demonstrating its fundamental role in bacterial adaptive evolution under antibiotic selection pressure. These findings redefine our understanding of β-lactamase evolution by revealing the structural plasticity of KPC enzymes and its contribution to antimicrobial resistance. They highlight the urgent need for integrated genomic surveillance and the development of next-generation β-lactamase inhibitors capable of countering this dynamic and convergent resistance mechanism.

## Contributors

BWZ and ZCZ conceived and supervised the study, critically revised the manuscript, and made the final decision to submit. HWY, RSL, and JS contributed equally to this work; they conducted the primary experimental investigations (including molecular, genomic, and phenotypic analyses described in the "Methods and materials" section), performed bioinformatics analysis, contributed to data interpretation, verified underlying data, and drafted the manuscript. XJL, and HX contributed to the experimental work and data analysis as members of the core laboratory team. WY, JFZ, and ZCZ contributed to clinical sample and/or data acquisition and clinical interpretation. TXH, KW, and LG contributed to specific analyses, provided resources, or offered intellectual input from their institution. All authors reviewed, read, and approved the final manuscript, had access to study data, and shared final responsibility for the decision to submit.

## Data sharing statement

The complete sequence of all strains has been submitted to GenBank under with BioProject number PRJNA1204189. In addition, the *bla*_KPC-263_ gene was deposited in the NCBI database under accession no. PV219192.

## Declaration of interests

All authors declare no conflicts of interest.
