# Supplementary material for: Unravelling tandem repeat-mediated mutagenesis drive rapid diversification of KPC enzymes: emergence of blaKPC-263 and enhanced resistance to ceftazidime-avibactam
Source: eBioMedicine. 2025 Oct 23;121:105979. doi: 10.1016/j.ebiom.2025.105979 (PMC12593533; doi:10.1016/j.ebiom.2025.105979)
Supplement: Supplementary Material 1 [file mmc1.docx]

**Supplemental material**

**Unraveling Tandem Repeat-Mediated Mutagenesis Drive Rapid Diversification of KPC Enzymes: Emergence of bla_KPC-263_ and Enhanced Resistance to Ceftazidime-Avibactam**

Haowei Ye^a,#^, Ruishan Liu^b,#^, Jie Shen^c,#^, Wei Yang^d^, Tongxi Hu^e^, Xiaojing Liu^a,f^, Kun Wang^e^, Lu Gong^e^, Hao Xu^a^, Junfei Zhu^g^, Zhencang Zheng^g,*^, Beiwen Zheng^a,f,h,**^

*^a^State Key Laboratory for Diagnosis and Treatment of Infectious Diseases, National Clinical Research Centre for Infectious Diseases, Collaborative Innovation Centre for Diagnosis and Treatment of Infectious Diseases, The First Affiliated Hospital, Zhejiang University School of Medicine, Hangzhou, China.*

*^b^Department of Critical Care Medicine, Second Affiliated Hospital, Zhejiang University School of Medicine, Hangzhou, China.*

*^c^Department of Medical Oncology, The First Affiliated Hospital, College of Medicine, Zhejiang University, Hangzhou, China.*

*^d^Department of Neurosurgery, The First Affiliated Hospital, College of Medicine, Zhejiang University, Hangzhou, China.*

*^e^School of Basic Medical Sciences, Zhejiang Chinese Medical University, Hangzhou, China.*

*^f^Jinan Microecological Biomedicine Shandong Laboratory, Jinan, China.*

*^g^Department of Respiratory and Critical Care Medicine, Taizhou Central Hospital, Taizhou, China*

*^h^Yuhang Institute for Collaborative Innovation and Translational Research in Life Sciences and Technology, Hanghzou, China*

**Figure S1.** Retention of IncFll/IncR plasmid in *K. pneumoniae* 72478/ *K. pneumoniae* 73003 during 15-day passages. Error bars represent standard deviation from three replicates. The error bars represent the 95% confidence interval of binomial ratios

**Figure S2.** Amino acid sequence alignment of OmpK36_72478, OmpK36_73003 and wild-type OmpK36

**Figure S3.** Comparison of *bla*_KPC_ gene copy numbers among different strains. The error bars represent the 95% confidence interval of binomial ratios.

**
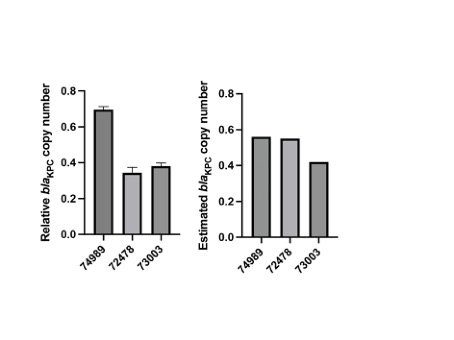
**

**Table S1** Primers involved in this study

| **Target** | **Primer name** | **Sequence （5'-3')** |
| --- | --- | --- |
| *bla*_KPC_ | KPC_N- F | ATGTCACTGTATCGCCGTCTAGT |
|  | KPC_N- R | TTACTGCCCGTTGACGCCCAA |
| *pgi*(for Qpcr) | Qpcr_pgi - F | TTCATCGCTCCGGCTATCAC |
|  | Qpcr_pgi - R | CCGGGTCTTTACCCTGATCG |
| *bla*_KPC_(for Qpcr) | Qpcr-KPC-F | CGCCGTCTAGTTCTGCTGTC |
|  | Qpcr-KPC-R | CCGCCAAAGTCCTGTTCGAG |

**Table S2** Whole-genome sequencing data of all strains

| **Strains** | **MLST** | **Sequence** | **Size(bp)** | **Plasmid Type** | **Resistance genes** | **Virulence**  **genes** |
| --- | --- | --- | --- | --- | --- | --- |
| 70251 | ST11 | Chromosome | 5,481,254 | \ | *oqxB*, *oqxA*, *bla*_SHV-182_, *fosA6* | *fyuA, ybtA/E/T/U/P/Q/X/S, irp1/2, ompA, entA/B, fepC,*  *yagV/X/Y/Z/W/K* |
|  |  | pMDR-Vir-70251 | 155,209 | IncFIB/II | *dfrA17*, *aadA5*, *sul1*, *mph(A)*, *sul2*, *aph(3'')-Ib*, *aph(6)-Id*, *tet(A)* | *iroB/C/D/E/N* |
|  |  | pKPC-70251 | 128,770 | IncR/FII | *bla*_CTX-M-65_, *fosA3*, *bla*_TEM-1B_, *rmtB*, *bla*_SHV-12_, *bla*_KPC-2_, *catA2* | \ |
|  |  | pCol-1-70251 | 11,970 | ColRNAI | \ | \ |
|  |  | pCol-2-70251 | 5,596 | ColRNAI | \ | \ |
| 70265 | ST11 | Chromosome | 5,481,256 | \ | *oqxB*, *oqxA*, *bla*_SHV-182_, *fosA6* | *fyuA, ybtA/E/T/U/P/Q/X/S, irp1/2, ompA, entA/B, fepC,*  *yagV/X/Y/Z/W/K* |
|  |  | pMDR-Vir-70265 | 155,203 | IncFIB/II | *dfrA17*, *aadA5*, *sul1*, *mph(A)*, *sul2*, *aph(3'')-Ib*, *aph(6)-Id*, *tet(A)* | *iroB/C/D/E/N* |
|  |  | pKPC-70265 | 128,770 | IncR/FII | *bla*_CTX-M-65_, *fosA3*, *bla*_TEM-1B_, *rmtB*, *bla*_SHV-12_, *bla*_KPC-2_, *catA2* | \ |
|  |  | pCol-1-70265 | 11,970 | ColRNAI | \ | \ |
|  |  | pCol-2-70265 | 5,596 | ColRNAI | \ | \ |
| 70378 | ST11 | Chromosome | 5,481,436 | \ | *oqxB*, *oqxA*, *bla*_SHV-182_, *fosA6* | *fyuA, ybtA/E/T/U/P/Q/X/S, irp1/2, ompA, entA/B, fepC,*  *yagV/X/Y/Z/W/K* |
|  |  | pMDR-Vir-70378 | 155,209 | IncFIB/II | *dfrA17*, *aadA5*, *sul1*, *mph(A)*, *sul2*, *aph(3'')-Ib*, *aph(6)-Id*, *tet(A)* | *iroB/C/D/E/N* |
|  |  | pKPC-70378 | 128,775 | IncR/FII | *bla*_CTX-M-65_, *fosA3*, *bla*_TEM-1B_, *rmtB*, *bla*_SHV-12_, *bla*_KPC-2_, *catA2* | \ |
|  |  | pCol-1-70378 | 11,970 | ColRNAI | \ | \ |
|  |  | pCol-2-70378 | 5,596 | ColRNAI | \ | \ |
| 70691 | ST11 | Chromosome | 5,480,626 | \ | *oqxB*, *oqxA*, *bla*_SHV-187_, *fosA6* | *fyuA, ybtA/E/T/U/P/Q/X/S, irp1/2, ompA, entA/B, fepC,*  *yagV/X/Y/Z/W/K* |
|  |  | pMDR-Vir-70691 | 155,209 | IncFIB/II | *dfrA17*, *aadA5*, *sul1*, *mph(A)*, *sul2*, *aph(3'')-Ib*, *aph(6)-Id*, *tet(A)* | *iroB/C/D/E/N* |
|  |  | pKPC-70691 | 128,757 | IncR/FII | *bla*_CTX-M-65_, *fosA3*, *bla*_TEM-1B_, *rmtB*, *bla*_SHV-12_, *bla*_KPC-2_, *catA2* | \ |
|  |  | pCol-1-70691 | 11,970 | ColRNAI | \ | \ |
|  |  | pCol-2-70691 | 5,596 | ColRNAI | \ | \ |
| 70714 | ST11 | Chromosome | 5,481,251 | \ | *oqxB*, *oqxA*, *bla*_SHV-182_, *fosA6* | *fyuA, ybtA/E/T/U/P/Q/X/S, irp1/2, ompA, entA/B, fepC,*  *yagV/X/Y/Z/W/K* |
|  |  | pKPC-70714 | 128,724 | IncFIB/II | *bla*_CTX-M-65_, *fosA3*, *bla*_TEM-213_, *rmtB*, *bla*_SHV-12_, *bla*_KPC-2_, *catA2* | \ |
|  |  | pCol-1-70714 | 11,970 | ColRNAI | \ | \ |
|  |  | pCol-2-70714 | 5,596 | ColRNAI | \ | \ |
| 70737 | ST11 | Chromosome | 5,407,944 | \ | *oqxB*, *oqxA*, *bla*_SHV-182_, *fosA6* | *fyuA, ybtA/E/T/U/P/Q/X/S, irp1/2, ompA, entA/B, fepC,*  *yagV/X/Y/Z/W/K* |
|  |  | pKPC-70737 | 126,679 | IncR/FII | *bla*_CTX-M-65_, *bla*_TEM-213_, *rmtB*, *bla*_SHV-12_, *bla*_KPC-2_, *catA2* | \ |
|  |  | pCol-1-70737 | 11,970 | ColRNAI | \ | \ |
|  |  | pCol-2-70737 | 5,596 | ColRNAI | \ | \ |
| 71003 | ST11 | Chromosome | 5,481,436 | \ | *oqxB*, *oqxA*, *bla*_SHV-182_, *fosA6* | *fyuA, ybtA/E/T/U/P/Q/X/S, irp1/2, ompA, entA/B, fepC,*  *yagV/X/Y/Z/W/K* |
|  |  | pKPC-71003 | 128,747 | IncR/FII | *bla*_CTX-M-65_, *fosA3*, *bla*_TEM-1B_, *rmtB*, *bla*_SHV-12_, *bla*_KPC-2_, *catA2* |  |
|  |  | pCol-1-71003 | 11.970 | ColRNAI |  |  |
|  |  | pCol-2-71003 | 5,596 | ColRNAI |  |  |
| 72478 | ST11 | Chromosome | 5,411,360 | \ | *oqxB*, *oqxA*, *bla*_SHV-182_, *fosA6* | *fyuA, ybtA/E/T/U/P/Q/X/S, irp1/2, ompA, entA/B, fepC,*  *yagV/X/Y/Z/W/K* |
|  |  | pKPC-72478 | 128,775 | IncR/FII | *bla*_CTX-M-65_, *fosA3*, *bla*_TEM-1B_, *rmtB*, *bla*_SHV-12_, *bla*_KPC-263_, *catA2* | \ |
|  |  | pCol-1-72478 | 11,970 | ColRNAI | \ | \ |
|  |  | pCol-2-72478 | 5,596 | ColRNAI | \ | \ |
| 72975 | ST11 | Chromosome | 5,481,261 | \ | *oqxB*, *oqxA*, *bla*_SHV-182_, *fosA6* | *fyuA, ybtA/E/T/U/P/Q/X/S, irp1/2, ompA, entA/B, fepC,*  *yagV/X/Y/Z/W/K* |
|  |  | pMDR-Vir-72975 | 155,246 | IncFIB/II | *dfrA17*, *aadA5*, *sul1*, *mph(A)*, *sul2*, *aph(3'')-Ib*, *aph(6)-Id*, *tet(A)* | *iroB/C/D/E/N* |
|  |  | pKPC-72975 | 126,640 | IncR/FII | *bla*_CTX-M-65_, *bla*_TEM-1B_, *rmtB*, *bla*_SHV-12_, *bla*_KPC-2_, *catA2* | \ |
|  |  | pCol-1-72975 | 11,970 | ColRNAI | \ | \ |
|  |  | pCol-2-72975 | 5,596 | ColRNAI | \ | \ |
| 73003 | ST11 | Chromosome | 5,412,029 | \ | *oqxB*, *oqxA*, *bla*_SHV-182_, *fosA6* | *fyuA, ybtA/E/T/U/P/Q/X/S, irp1/2, ompA, entA/B, fepC,*  *yagV/X/Y/Z/W/K* |
|  |  | pKPC-73003 | 128,724 | IncR/FII | *bla*_CTX-M-65_, *fosA3*, *bla*_TEM-207_, *rmtB*, *bla*_SHV-12_, *bla*_KPC-90_, *catA2* | \ |
|  |  | pCol-1-73003 | 11,970 | ColRNAI | \ | \ |
|  |  | pCol-2-73003 | 5,596 | ColRNAI | \ | \ |
| 73116 | ST11 | Chromosome | 5,482,454 | \ | *oqxB*, *oqxA*, *bla*_SHV-182_, *fosA6* | *fyuA, ybtA/E/T/U/P/Q/X/S, irp1/2, ompA, entA/B, fepC,*  *yagV/X/Y/Z/W/K* |
|  |  | pKPC-73116 | 126,614 | IncR/FII | *bla*_CTX-M-65_, *bla*_TEM-1B_, *rmtB*, *bla*_SHV-182_, *bla*_KPC-2_, *catA2* | \ |
|  |  | pCol-1-73116 | 11,970 | ColRNAI | \ | \ |
|  |  | pCol-2-73116 | 5,596 | ColRNAI | \ | \ |
| 73264 | ST11 | Chromosome | 5,482,455 | \ | *oqxB*, *oqxA*, *bla*_SHV-182_, *fosA6* | *fyuA, ybtA/E/T/U/P/Q/X/S, irp1/2, ompA, entA/B, fepC,*  *yagV/X/Y/Z/W/K* |
|  |  | pMDR-Vir-73264 | 155,080 | IncFIB/II | *dfrA17*, *aadA5*, *sul1*, *mph(A)*, *sul2*, *aph(3'')-Ib*, *aph(6)-Id*, *tet(A)* | *iroB/C/D/E/N* |
|  |  | pKPC-73264 | 128,649 | IncR/FII | *bla*_CTX-M-65_, *fosA3*, *bla*_TEM-1B_, *rmtB*, *bla*_SHV-12_, *bla*_KPC-2_, *catA2* | \ |
|  |  | pCol-1-73264 | 11,970 | ColRNAI | \ | \ |
|  |  | pCol-2-73264 | 5,596 | ColRNAI | \ | \ |
| 73780 | ST11 | Chromosome | 5,481,586 | \ | *oqxB*, *oqxA*, *bla*_SHV-182_, *fosA6* | *fyuA, ybtA/E/T/U/P/Q/X/S, irp1/2, ompA, entA/B, fepC,*  *yagV/X/Y/Z/W/K* |
|  |  | pKPC-73780 | 126,679 | IncR/FII | *catA2*, *bla*_CTX-M-65_, *bla*_TEM-1B_, *rmtB*, *bla*_KPC-2_, *bla*_SHV-12_ | \ |
|  |  | pCol-1-73780 | 11,970 | ColRNAI | \ | \ |
|  |  | pCol-2-73780 | 5,596 | ColRNAI | \ | \ |
| 74451 | ST11 | Chromosome | 5,482,477 | \ | *oqxB*, *oqxA*, *bla*_SHV-182_, *fosA6* | *fyuA, ybtA/E/T/U/P/Q/X/S, irp1/2, ompA, entA/B, fepC,*  *yagV/X/Y/Z/W/K* |
|  |  | pKPC-74451 | 126,679 | IncR/FII | *bla*_CTX-M-65_, *bla*_TEM-1B_, *rmtB*, *bla*_SHV-12_, *bla*_KPC-2_, *catA2* | \ |
|  |  | pCol-1-74451 | 11,970 | ColRNAI |  | \ |
|  |  | pCol-2-74451 | 5,596 | ColRNAI |  | \ |
| 74989 | ST11 | Chromosome | 5,482,211 | \ | *oqxB*, *oqxA*, *bla*_SHV-182_, *fosA6* | *fyuA, ybtA/E/T/U/P/Q/X/S, irp1/2, ompA, entA/B, fepC,*  *yagV/X/Y/Z/W/K* |
|  |  | pKPC-74989 | 126,676 | IncR/FII | *bla*_CTX-M-65_, *bla*_TEM-1B_, *rmtB*, *bla*_SHV-12_, *bla*_KPC-2_, *catA2* | \ |
|  |  | pCol-1-74989 | 11,970 | ColRNAI | \ | \ |
|  |  | pCol-2-74989-3 | 5,596 | ColRNAI | \ | \ |

**Table S3** Predicted binding affinities of KPC-2, KPC-263, and KPC-90 variants with ceftazidime and avibactam based on molecular docking simulations.

| **Protein** | **Mutation profile** | **Ligands** | **Affinity (kcal/mol)** |
| --- | --- | --- | --- |
| KPC-2 | Wild-type | Ceftazidime | -7.4 |
|  |  | Avibactam | -6.3 |
| KPC-263 | Ins_238_G | Ceftazidime | -6.9 |
|  |  | Avibactam | -5.8 |
| KPC-90 | Ins_179-180_TY | Ceftazidime | -6.7 |
|  |  | Avibactam | -5.8 |
